# Serial QuantiFERON-TB Gold testing in patients with psoriasis treated with ustekinumab

**DOI:** 10.1371/journal.pone.0184178

**Published:** 2017-09-08

**Authors:** Chuan-Yu Hsiao, Hsien-Yi Chiu, Ting-Shun Wang, Tsen-Fang Tsai

**Affiliations:** 1 Department of Dermatology, National Taiwan University Hospital and National Taiwan University College of Medicine, Taipei, Taiwan; 2 Department of Dermatology, National Taiwan University Hospital Hsin-Chu Branch, Hsinchu, Taiwan; 3 Department of Dermatology, National Taiwan University Hospital, Yun-Lin Branch, Dou-Liou, Taiwan; Katholieke Universiteit Leuven Rega Institute for Medical Research, BELGIUM

## Abstract

**Background:**

There is increasing concern about the risk of latent tuberculosis infection (LTBI) reactivation during the use of biologics for psoriasis. Although ustekinumab had been documented with low risk of tuberculosis, the long-term follow-up of LTBI as determined by QuantiFERON-TB Gold (QFT-G) testing in patients treated with ustekinumab is limited.

**Objectives:**

This study aims to use serial QFT-G testing as a screening method for detecting LTBI in patients with psoriasis from an intermediate tuberculosis burden country.

**Methods:**

This retrospective review investigated 134 psoriatic patients in whom ustekinumab was prescribed for at least one year between 2010 and 2016 in National Taiwan University Hospital. All patients underwent annular QFT-G testing during ustekinumab therapy.

**Results:**

Among the 134 enrolled patients, baseline LTBI rate was 13.4% (18/134). Indeterminate QFT-G result was noted in 5.2% (7/134) of patients and 71.4% (5/7) of them turn to be QFT-G negative during the next testing. 81.3% (109/134) of patients had a negative QFT-G at baseline and the seroconversion rate was 7.3% (8/109) in the serial QFT-G. All the patients in the conversion group were referred to a pulmonologist for evaluation and 81.5% (22/27) of them underwent chemoprophylactic therapy while on ustekinumab. No active TB infection was noted during further follow-up with or without chemoprophylaxis.

**Conclusions:**

This study revealed that psoriatic patients receiving long-term ustekinumab therapy had a low QFT-G conversion rate (7.3%). The clinical significance of QFT-G conversion remains controversial and needs larger scale trials to investigate.

## Introduction

Biologics have been increasingly used in the treatment of moderate to severe psoriasis due to their efficacy and safety compared to pre-existing treatment modalities. Anti-tumor necrosis factors (TNF), and ustekinumab (a monoclonal antibody against the p40 subunit of IL-12 and IL-23) are currently the most commonly used biologics for psoriasis. Biologics are also considered to be safer than conventional systemic agents except for increased granulomatous infections, mainly tuberculosis (TB). Screening and monitoring for both active and latent TB infection (LTBI) before and during treatment with TNF-blockers are considered to be the standard of care [1–6]. Among the different methods of LTBI detection, whole blood interferon-γ release assays (IGRAs) offer better sensitivity and specificity than tuberculin skin test (TST) in detecting LTBI (sensitivity 89% vs. 74%; specificity 98% vs. 81%) [7, 8], especially in subjects who had received Bacilli Calmette-Guerin (BCG) vaccination previously [9,10]. There are two commercially available IGRAs, T-Spot and QuantiFERON-TB Gold In-Tube test (QFT-GIT; Cellestis Limited, Carnegie, Victoria, Australia), and the latter test is more commonly used due to its lower cost and ease to perform.

The validity of IGRAs in screening LTBI before initiating TNF-α antagonists had been well documented [11–14]. The prevalence of LTBI varies according to countries, and was 12/110 (11%) [15] and 10/101 (10%) [16] in Taiwan among TNF-blockers users for psoriasis. The optimal timing or need of IGRAs retest has not been standardized in otherwise asymptomatic biologic users, and seroconversion rate may be affected by the background TB prevalence, frequency of IGRAs retest, patient demographics, types of underlying diseases, types of biologics and concomitant medications. Serial QFT tests have revealed annual seroconversion rate between 7%~ 18% in TNF-blockers users in psoriasis [17–20], and it was 14.29% in Taiwan [16]. Risks of TB and TBLI reactivation are considered to be lower for using ustekinumab compared to TNF-blockers. However, there are limited studies regarding the serial IGRAs results among patients who received ustekinumab therapy. This study aimed to evaluate the serial QFT-GIT results in patients receiving long-term ustekinumab treatment in Taiwan, a country with an intermediate prevalence of tuberculosis.

## Materials and methods

### Patients

The study was approved by the local investigational research bureau of National Taiwan University Hospital. Screening for LTBI is mandatory before and during biologics use for psoriasis in Taiwan. According to local risk management requirement of all biologics used for psoriasis, QFT-GIT has to be performed once a year in routine practice. Both serial IGRA testing and regular chest X-ray examination were performed. We retrospectively enrolled all psoriatic patients treated with ustekinumab from May 2010 to September 2016 in National Taiwan University Hospital, Taipei and Hsin-Chu, Taiwan. All patients who used ustekinumab and have received quantiferon test at least twice separated by at least one year during the study period were included. The time span between serial QFT for a single patient was at least 12 months respectively. We reviewed the medial records of these patients to evaluate clinical diagnosis, demographic information, medical history and laboratory data.

### QuantiFERON-TB Gold In-Tube assay for latent tuberculosis infection

QFT-GIT was performed in all patients with ustekinumab use. Antigen with peptide cocktail simulating the proteins ESAT-6, CFP-10 and TB7.7 were used in the test. The interferon-γ (IFN-γ) values were calculated by subtracting the obtained value with nil antigens. A positive result was defined as IFN-γ≥ 0.35 IU/ml and positive control value (IFN-γ of mitogen minus nil antigens) ≥ 0.5 IU/ml. A negative result was defined as 0 < IFN-γ< 0.35 IU/ml and positive control (mitogen) value ≥ 0.5 IU/ml. Indeterminate result was defined as IFN-γ of nil antigen >8 IU/ml or positive control value < 0.5 IU/ml. Conversion of IGRA was defined as a negative IGRA at baseline and a positive IGRA at follow-up, while reversion of IGRA was defined as a positive IGRA at baseline and negative IGRA at follow-up.

### Treatment and follow-up of LTBI

The diagnosis of LTBI in our study was defined as a positive QFT-GIT result and a negative chest X-ray or microbiological assay. All patients diagnosed with LTBI were referred to pulmonologists for evaluation and underwent further survey and chemoprophylactic therapy if recommended. The recommendation of tuberculosis screening and treatment in Taiwan was originally based on anti-TNF agents. However, no modification was made after the introduction of ustekinumab. For anti-TNF agents, an at least 4-week treatment with isoniazid (INH) before anti-TNF agent use was suggested in cases of latent TB detected by either IRGAs or tuberculin skin test. A 9-month INH is needed for latent TB. Since the tuberculosis risk of ustekinumab is supposed to be much lower than anti-TNF agents, and there is a risk of INH resistance for tuberculosis, the infection specialists will decide the appropriateness of INH prophylaxis according to their risk assessment results based on clinical symptoms, chest X ray findings, exposure sources, and sputum culture results. In clinical practice, ustekinumab is usually prescribed at the same time as INH initiation in cases without any other evidence of latent tuberculosis. In fact, in the registration trial of ustekinumab for psoriasis in Taiwan, ustekinumab was approved to be given at the same day as INH initiation [21].

### Statistical analysis

Statistical analysis and graphs were done with standard spreadsheet software program using Microsoft Excel 2010 (Microsoft Corporation, Seattle, WA, USA) and Fisher exact test. Statistical significant was defined as p value < 0.05.

## Results

### Patient characteristics

We enrolled 134 patients to this study in total. Of these patients, the mean age was 47.16 years (standard deviation((SD)) 13.14 years) and the male-to female ratio was 101/33. The mean follow-up months were 20.5 (SD 10.52) and 42 patients (32.1%) also had psoriatic arthritis. Most patients were biologics naïve, 24 patients (17.9%) had received at least 6 months of etanercept and 23 patients (17.2%) had received at least 6 months of adalimumab. During ustekinumab treatment, 17 patients (12.7%) also received concomitant immunosuppressant therapy or other biological agents. Among these agents, methotrexate was the most commonly used drug (n = 12, 9%). The demographic features are presented in Table 1.

**Table 1:** Dermographic features.

**Age (years)**	**47.16 ± 13.14**^a^
**Sex, n (%)**	**Man: 101 (75.4)**
	**Woman: 33 (24.6)**
**With psoriatic arthritis, n (%)**	**42 (32.1)**
**Mean follow-up months**	**20.5 ± 10.52**^a^
**Previous biological treatment, n (%)**	**Etanercept: 24 (17.9)**
	**Adalimumab: 23 (17.2)**
**Combined treatment, n (%)**	**Methotrexate: 12 (9)**
	**Etanercept: 4 (3)**
	**Adalimumab: 1(0.7)**

^**a**^Standard deviation

### Serial QuantiFERON-TB Gold In-Tube assay results

**Fig 1:**
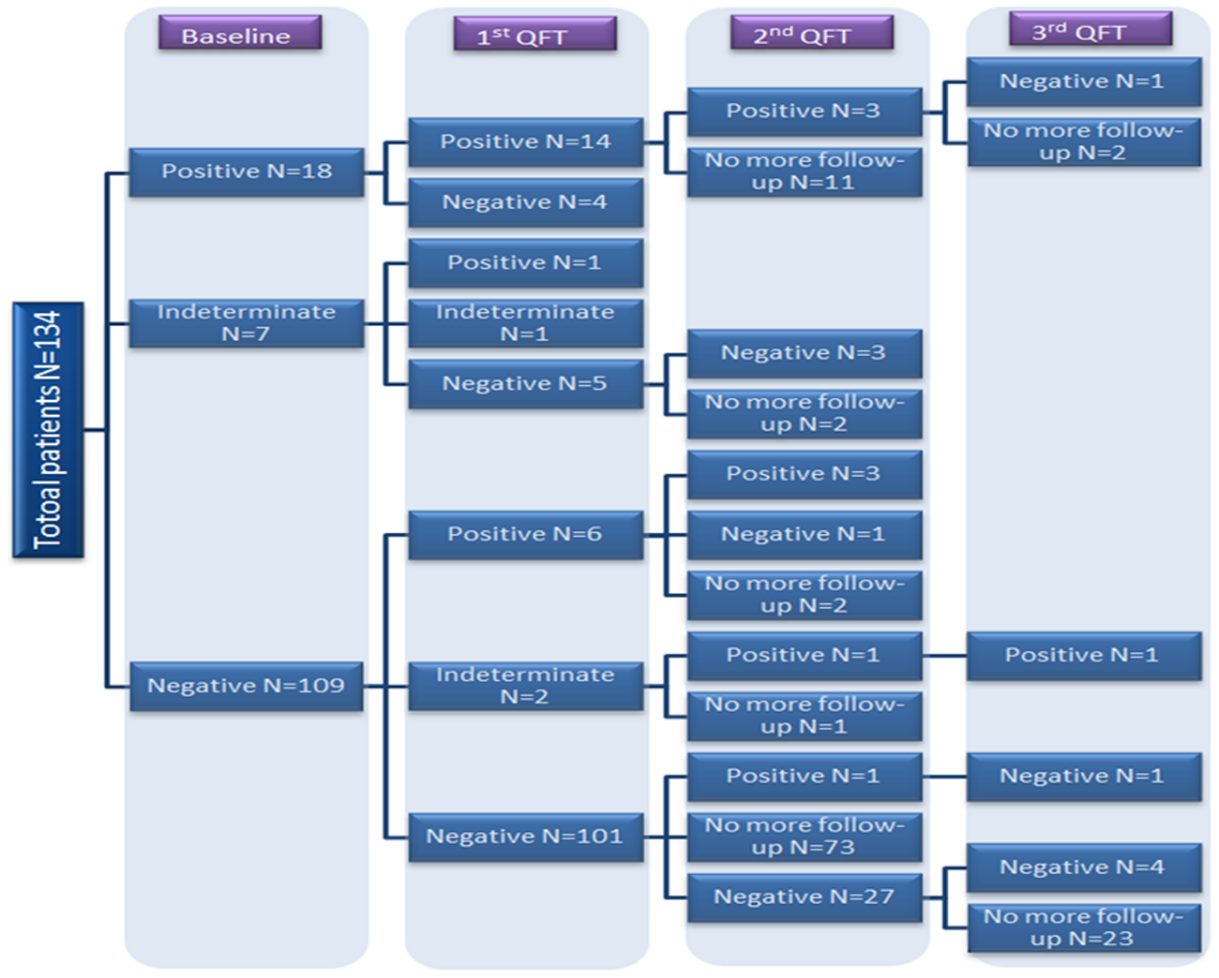
Flowchart distribution of QantiFERON Gold In-Tube test results.

**Fig 2:**
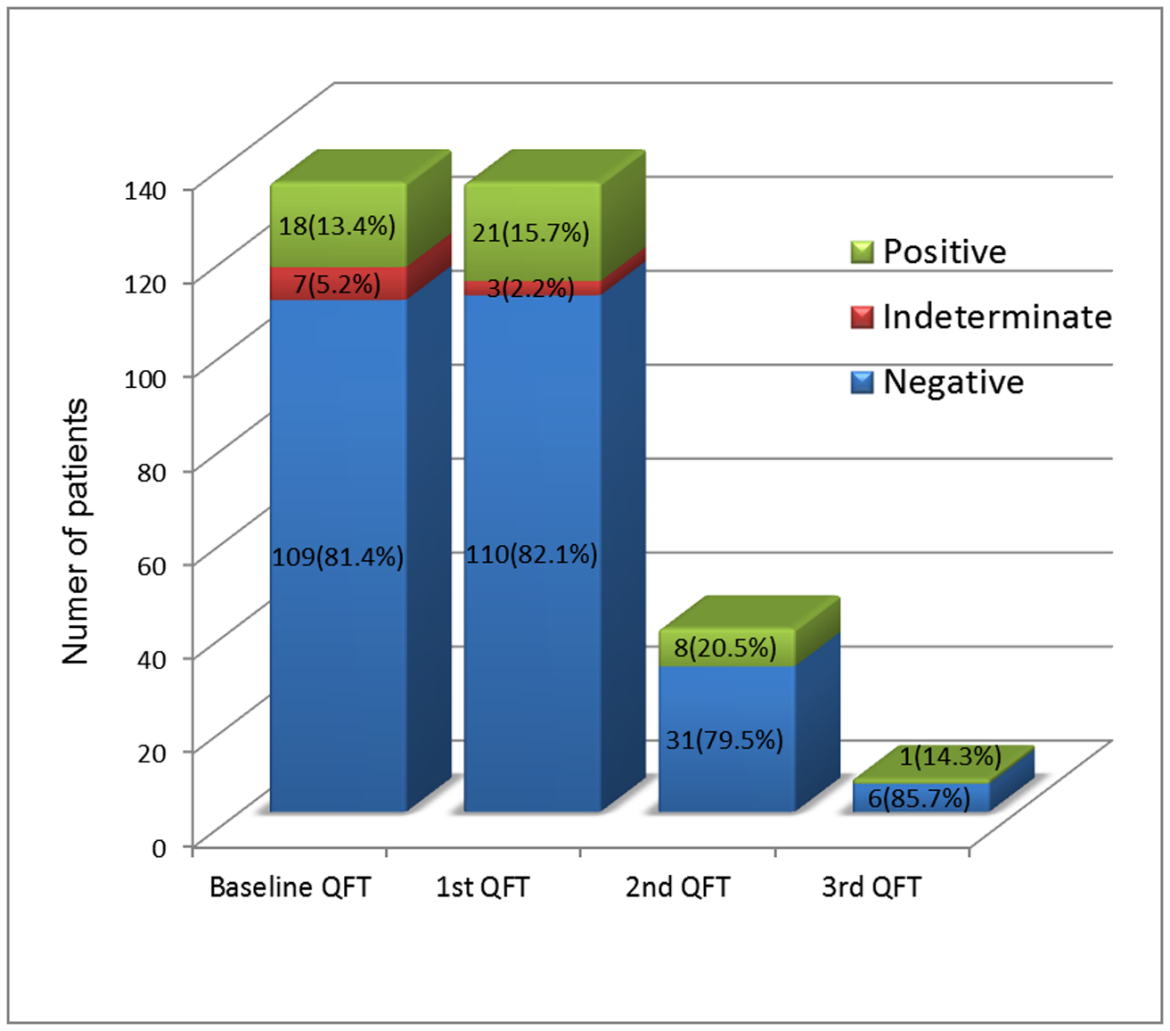
Results of serial QuantiFERON-TB Gold In-Tube tests.

**Fig 3:**
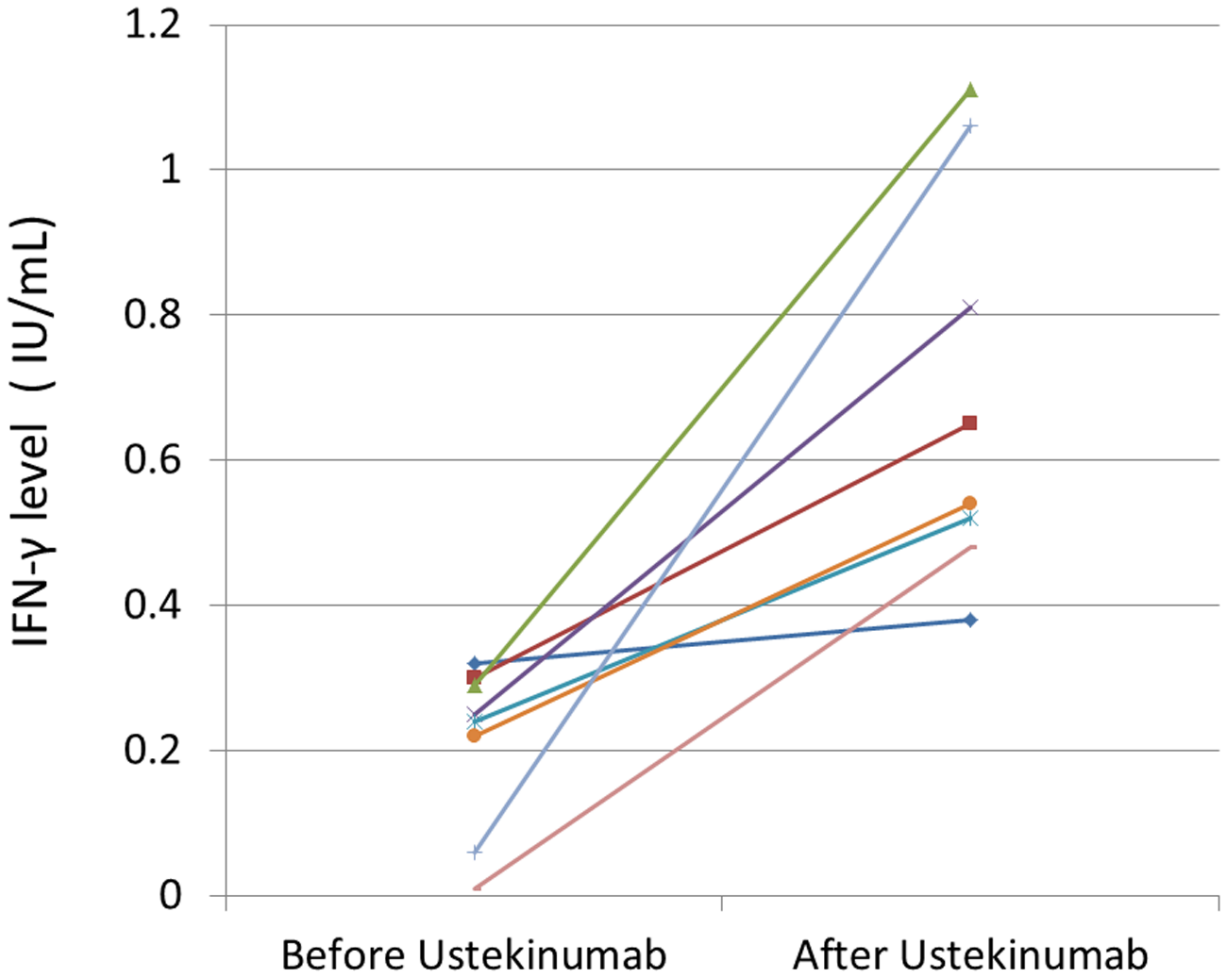
IFN-γ level before and after ustekinumab treatment in the conversion group.

The serial QFT-GIT assay results are summarized in the study flowchart (Fig 1) and histogram (Fig 2). All the patients had received baseline QFT and 2^nd^ QFT at least 12 months later. 39 patients (29.1%) had undergone 3^rd^ QFT and 7 patients (5.2%) had received 4^th^ QFT. The initial IGRA result was positive in 18 patients (13.4%), negative in 109 patients (81.4%) and indeterminate in 7 patients (5.2%). The conversion rate was 7.3% (8/109) in the serial QFT. Six patients had QFT seroconversion at the 2^nd^ QFT follow-up while 2 patients had seroconversion at the 3^rd^ QFT follow-up. The longitudinal IFN-γ level changes before and after ustekinumab treatment in the seroconversion group were shown in Fig 3.

We divided patient data according to (I) patients receiving only ustekinumab (n = 117) and (II) patients receiving another treatment as well (n = 17). The seroconversion patient number in each group was 7 and 1 respectively and showed no statistically significant difference (Table 2).

**Table 2:** Seroconversion patient number from (I) patients receiving only ustekinumab(II) patients receiving ustekinumab along with other treatment.

	Patients receiving only ustekinumab (number = 117)	Patients receiving ustekinumab along with other treatment as well (number = 17)	p value
**Seroconversion patient number**	7	1	1

Patients were also divided into two subgroups: (I) biologic-naïve group who had never received biologics agents before using ustekinumab (n = 97) (II) non-biologic-naïve group (n = 37). The seroconversion patient number in each group was 5 and 2 respectively and revealed no statistically significant difference (Table 3).

**Table 3:** Seroconversion patient number from (I) biologics-naïve patients (II) non-biologics-naïve patients.

	Biologics-naïve patients (number = 97)	Non-biologics-naïve patients (number = 37)	p value
**Seroconversion patient number**	5	2	1

Totally 27 patients were diagnosed with LTBI in our study. All were referred to pulmonologists for evaluation. Among them, one patient had poor compliance and was lost to follow-up afterwards. 16 patients (59.2%) had received 9-month course of prophylactic therapy with 300 mg of daily isoniazid (INH) and had been follow-up for an average of 17.2 months. 5 patients (18.5%) had only received 1~3 months of INH prophylactic due to nausea, vomiting and elevated liver enzyme and received follow-up for an average of 11.4 months. The rest 5 patients (18.5%) didn’t receive prophylactic therapy after pulmonologists’ evaluation and had been followed up for an average of 29.2 months. During follow-up, no active TB infection was detected in our study.

## Discussion

Biologic agents had become increasingly used in treating moderate to severe psoriasis and psoriatic arthritis since 2002. Opportunistic infections were one of the most important adverse effects during usage of biologic agents. Patients treated with TNF-blockers have increased risk of LTBI reactivation and need to undergo regular TB screening [1]. Regular screening for LTBI during TNF-blockers or ustekinumab treatment had been listed into guidance in many professional dermatologic associations including American Academy of Dermatology (AAD), the Japanese Dermatology Association (JDA) and the European Academy of Dermatology and Venereology (EADV) [5]. Among the screening methods, IGRA such as QFT-GIT was preferred due to higher sensitivity and specificity [7, 8]. Risks of TB and LTBI reactivation are generally considered lower for ustekinumab compared to TNF-blockers. While previous studies indicate that patients with inborn errors of IL-12/ 23- IFN-γ-mediated immunity area are at higher risk for developing TB infection [22], cases of TB reactivation or infection during ustekinumab use remain limited. Across five phase III clinical trials of ustekinumab-treated patients with psoriasis, no cases of LTBI reactivation was observed in patients receiving concomitant INH prophylaxis [23]. However, reactivation of LTBI during ustekinumab and low dose oral steroid treatment had been reported in a patient who underwent prophylactic therapy with isoniazid [24].

The seroconversion rate from our study was 7.3%, which is lower than previous study (14.29%) conducted among psoriasis patients treated with anti-tumor necrosis factors in Taiwan [16]. Accuracy of IGRAs during biological therapy has been disputed, especially for patients undergoing anti-TNF blockers. For example, it was found that the development of positive QFT-GIT, observed in 8% of patients treated with infliximab, was not associated with pulmonary or extra-pulmonary tuberculosis [25]. In the other study, adalimumab (a TNF blocker) was found to significantly reduce the IFN-γ levels, potentially causing false negative QF results in a dose-dependent manner [26]. As for ustekinumab, an IL12/IL23 blocker, there are no prior reports of serial quantiferon results in patients receiving ustekinumab for psoriasis. Although, a prior study showed that treatment with ustekinumab resulted in no changes of serum TNF levels [27], maintenance of pulmonary Th1 effector function in chronic tuberculosis requires persistent IL-12 production [28]. Thus, we suspect the QFT seroconversion rate might reflect either a background tuberculosis exposure or from a prior false negative QFT test result at baseline before ustekinumab treatment, such as in the case report of latent tuberculosis reactivation with quantiferon conversion [29]. Weather the fluctuation of IFN-γ level correlated with clinical significance still remained controversial. Some reported that there is a biphasic emergence of TB infection in patients receiving TNF-blockers. Persistently high levels of released IFN-γ (during the first 3 months) or QFT conversion (after 20–24 months) strongly indicate the development of active tuberculosis in patients undergoing long-term anti-TNFα therapy [30]. Some advocate that dynamic changes occurring with serial IFN-γ release assay testing in patients treated with biologic therapy do not correlate with clinical outcome [31]. Scrivo et al proposed a concept of “zone of uncertainty” and re-defined the cut off value of QFT positive result. The zone of uncertainty was defined in the range of 0.2–0.5 IU/mL IFN-γ levels. Any results fluctuate within the uncertainty zone during repeated testing were considered doubtful conversions or doubtful reversions. As seen in Fig 3, some IFN-γ levels did fluctuate within the uncertainty zone. After excluding these doubtful conversions, seroconversion rate would be decreased to 3.7% in our study. In further study, Mancuso et al found that patients with negative TB responses near the cutoff (i.e., ≥0.25 but <0.35 IU/ml) were, 30 times more likely to convert to positive than those with responses further from the cutoff [32]. As the IGRAs are complicated laboratory tests that may require at least 126 measurements for a single test [33], the in-test variability may contribute to the zone of uncertainty. Use of this borderline zone may reduce the conversion numbers but would increase the number of patients with uncertain results. Further evaluation of risk, serial duplicate testing and long-term follow-up in clinical practice are needed to clarify the usefulness of the concept of “zone of uncertainty”.

The QF seroconversion rate might reflect either a background tuberculosis exposure or from a prior false negative QFT test result at baseline. A background tuberculosis exposure may differ significantly in different areas and diseases. Taiwan is an intermediate tuberculosis burden country. Although the prior reports show QF conversion rate to be between 7%~ 18% in TNF-blockers users in psoriasis [17–20], the 7.3% percent is still lower than the experience of TNF blockers for psoriasis in Taiwan [16]. Moreover, the LTBI rate in our study was 13.4% (18/134), similar to previous studies: 12/110 (11%) [15] and 10/101 (10%) [16] in Taiwan among TNF-blockers users for psoriasis. All the patients with LTBI were referred to pulmonologists for evaluation and most patients had underwent 1~9-month course of INH prophylactic therapy while 5 patients (18.5%) didn’t. During further follow-up, no patients from either INH prophylactic or non-prophylactic group developed active tuberculosis. Although chemoprophylactic therapy can efficiently decrease the LTBI reactivation rate in patients who underwent ustekinumab therapy [23], adverse effect such as liver toxicity and increased drug resistance had also been proposed. The possibility of pre-existing or newly emergent INH resistant *Mycobacterium tuberculosis* strains has also been considered. Thus INH monotherapy, although recommended, is not always prescribed by all referral pulmonologists, especially in low risk patients. Apart from IGRA, clinical vigilance which incorporates careful evaluation of the clinical symptoms and signs, the contact and exposure history, and the TB prevalence rate of the endemic area are needed to guide the final treatment decision.

There are several limitations in the study. First, this is a non-prospective study, which results in some variation in the exact QF retest time. However, most patients received annual tests according to the risk management requirement. Second, some patients received concomitant systemic therapy. However, methotrexate is mainly used and methotrexate has not been found to increase TB risk in prior study when used alone in psoriasis. Additionally, even with concomitant use of TNF blockers, none of the subjects developed tuberculosis in the study [34]. Third, the subjects who had long term follow-up beyond two years are still limited.

In summary, our study revealed that psoriatic patients had a lower QFT-GIT seroconversion rate (7.3%) after ustekinumab therapy compared to anti-tumor necrosis factors therapy. No active tuberculosis was detected during serial follow-up. However, a larger sample size with longer follow-up is still needed to fully assess the TB risks associated with ustekinumab therapy.

## Supporting information

S1 Dataset Primay data of enrolled patients. (XLSX)

S1 Checklist STROBE checklist. (DOC)

## References

[1] Keane J, Gershon S, Wise RP, Mirabile-Levens E, Kasznica J, Schwieterman WD, et al (2001) Tuberculosis associated with infliximab, a tumor necrosis factor alpha-neutralizing agent. N Engl J Med 345: 1098–1104. doi: 10.1056/NEJMoa011110 11596589 10.1056/NEJMoa011110

[2] Gómez-Reino JJ, Carmona L, Valverde VR, Mola EM, Montero MD. (2003) Treatment of rheumatoid arthritis with tumor necrosis factor inhibitors may predispose predispose to significant increase in tuberculosis risk: a multicenter active-surveillance report. Arthritis Rheum 48: 2122–2127. doi: 10.1002/art.11137 12905464 10.1002/art.11137

[3] Mohan AK, Coté TR, Block JA, Manadan AM, Siegel JN, Braun MM. (2004) Tuberculosis following the use of etanercept, a tumor necrosis factor inhibitor. Clin Infect Dis 39: 295–299. doi: 10.1086/421494 15306993 10.1086/421494

[4] Bongartz T, Sutton AJ, Sweeting MJ, Buchan I, Matteson EL, Montori V. (2006) Anti-TNF antibody therapy in rheumatoid arthritis and the risk of serious infections and malignancies: systematic review and meta-analysis of rare harmful effects in randomized controlled trials. JAMA 295: 2275–2285. doi: 10.1001/jama.295.19.2275 16705109 10.1001/jama.295.19.2275

[5] Ahn CS, Dothard EH, Garner ML, Feldman SR, Huang WW. (2015) To test or not to test? An updated evidence-based assessment of the value of screening and monitoring tests when using systemic biologic agents to treat psoriasis and psoriatic arthritis. J Am Acad Dermatol 73: 420–428.e1. doi: 10.1016/j.jaad.2015.06.004 26184440 10.1016/j.jaad.2015.06.004

[6] Sivamani RK, Goodarzi H, Garcia MS, Raychaudhuri SP, Wehrli LN, Ono Y, et al (2013) Biologic therapies in the treatment of psoriasis: a comprehensive evidence-based basic science and clinical review and a practical guide to tuberculosis monitoring. Clin Rev Allergy Immunol 44: 121–140. doi: 10.1007/s12016-012-8301-7 22311162 10.1007/s12016-012-8301-7

[7] Mori T, Sakatani M, Yamagishi F, Takashima T, Kawabe Y, Nagao K, et al (2004) Specific detection of tuberculosis infection: an interferon-gamma-based assay using new antigens. Am J Respir Crit Care Med 170: 59–64. doi: 10.1164/rccm.200402-179OC 15059788 10.1164/rccm.200402-179OC

[8] Lein AD, von Reyn CF, Ravn P, Horsburgh CR Jr, Alexander LN, Andersen P. (1999) Cellular immune responses to ESAT-6 discriminate between patients with pulmonary disease due to Mycobacterium avium complex and those with pulmonary disease due to Mycobacterium tuberculosis. Clin Diagn Lab Immunol 6: 606–609. 10391871 10.1128/cdli.6.4.606-609.1999 PMC95736

[9] Diel R, Loddenkemper R, Meywald-Walter K, Niemann S, Nienhaus A. (2008) Predictive value of a whole blood IFN-gamma assay for the development of active tuberculosis disease after recent infection with Mycobacterium tuberculosis. Am J Respir Crit Care Med 177: 1164–1170. doi: 10.1164/rccm.200711-1613OC 18276940 10.1164/rccm.200711-1613OC

[10] Gisondi P, Cazzaniga S, Chimenti S, Maccarone M, Picardo M, Girolomoni G, et al (2014) Latent tuberculosis infection in patients with chronic plaque psoriasis who are candidates for biological therapy. Br J Dermatol 171: 884–890. doi: 10.1111/bjd.13130 24863903 10.1111/bjd.13130

[11] Chen DY, Shen GH, Hsieh TY, Hsieh CW, Lan JL. (2008) Effectiveness of the combination of a whole-blood interferon-gamma assay and the tuberculin skin test in detecting latent tuberculosis infection in rheumatoid arthritis patients receiving adalimumab therapy. Arthritis Rheum 59: 800–806. doi: 10.1002/art.23705 18512714 10.1002/art.23705

[12] Pratt A, Nicholl K, Kay L. (2007) Use of the QuantiFERON TB Gold test as part of a screening programme in patients with RA under consideration for treatment with anti-TNF-alpha agents: the Newcastle (UK) experience. Rheumatology (Oxford) 46: 1035–1036. doi: 10.1093/rheumatology/kem064 17409126 10.1093/rheumatology/kem064

[13] Ponce de Leon D, Acevedo-Vasquez E, Alvizuri S, Gutierrez C, Cucho M, Alfaro J, et al (2008) Comparison of an interferon-gamma assay with tuberculin skin testing for detection of tuberculosis (TB) infection in patients with rheumatoid arthritis in a TB-endemic population. J Rheumatol 35: 776–781. 18398944

[14] Matulis G, Jüni P, Villiger PM, Gadola SD. (2008) Detection of latent tuberculosis in immunosuppressed patients with autoimmune diseases: performance of a Mycobacterium tuberculosis antigen-specific interferon gamma assay. Ann Rheum Dis 67: 84–90. doi: 10.1136/ard.2007.070789 17644549 10.1136/ard.2007.070789

[15] Chiu HY, Hsueh PR, Tsai TF. (2011) Clinical experience of QuantiFERON^®^ -TB Gold testing in patients with psoriasis treated with tumor necrosis factor blockers in Taiwan. Br J Dermatol 164: 553–559. doi: 10.1111/j.1365-2133.2010.10137.x 21083541 10.1111/j.1365-2133.2010.10137.x

[16] Cheng CY, Hui CY, Sindy Hu, Hsieh MH, Huang YH. (2015) Serial QuantiFERON-TB Gold In-Tube testing for psoriatic patients receiving antitumor necrosis factor-alpha therapy. Dermatol Sinica 33: 124–129.

[17] Kim KH, Lee SW, Chung WT, Kim BG, Woo KS, Han JY, et al (2011) Serial interferon-gamma release assays for the diagnosis of latent tuberculosis infection in patients treated with immunosuppressive agents. Korean J Lab Med 31: 271–278. doi: 10.3343/kjlm.2011.31.4.271 22016681 10.3343/kjlm.2011.31.4.271 PMC3190006

[18] Garcovich S, Ruggeri A, D'Agostino M, Ardito F, De Simone C, Delogu G, et al (2012) Clinical applicability of Quantiferon-TB-Gold testing in psoriasis patients during long-term anti-TNF-alpha treatment: a prospective, observational study. J Eur Acad Dermatol Venereol 26: 1572–1576. doi: 10.1111/j.1468-3083.2011.04220.x 21923840 10.1111/j.1468-3083.2011.04220.x

[19] Hatzara C, Hadziyannis E, Kandili A, Koutsianas C, Makris A, Georgiopoulos G, et al (2015) Frequent conversion of tuberculosis screening tests during anti-tumour necrosis factor therapy in patients with rheumatic diseases. Ann Rheum Dis 74: 1848–1853. doi: 10.1136/annrheumdis-2014-205376 24854354 10.1136/annrheumdis-2014-205376

[20] Bartalesi F, Goletti D, Spinicci M, Cavallo A, Attala L, Mencarini J, et al (2013) Serial QuantiFERON TB-gold in-tube testing during LTBI therapy in candidates for TNFi treatment. J Infect 66: 346–356. doi: 10.1016/j.jinf.2012.10.017 23103667 10.1016/j.jinf.2012.10.017

[21] Tsai TF, Ho JC, Song M, Szapary P, Guzzo C, Shen YK, et al (2011) Efficacy and safety of ustekinumab for the treatment of moderate-to-severe psoriasis: a phase III, randomized, placebo-controlled trial in Taiwanese and Korean patients (PEARL). J Dermatol Sci 63: 154–163. doi: 10.1016/j.jdermsci.2011.05.005 21741220 10.1016/j.jdermsci.2011.05.005

[22] Filipe-Santos O, Bustamante J, Chapgier A, Vogt G, de Beaucoudrey L, Feinberg J, et al (2006) Inborn errors of IL-12/23- and IFN-gamma-mediated immunity: molecular, cellular, and clinical features. Semin Immunol 18: 347–361. doi: 10.1016/j.smim.2006.07.010 16997570 10.1016/j.smim.2006.07.010

[23] Tsai TF, Ho V, Song M, Szapary P, Kato T, Wasfi Y, et al (2012) The safety of ustekinumab treatment in patients with moderate-to-severe psoriasis and latent tuberculosis infection. Br J Dermatol 167: 1145–1152. doi: 10.1111/j.1365-2133.2012.11142.x 22803615 10.1111/j.1365-2133.2012.11142.x

[24] Errichetti E, Piccirillo A. (2014) Latent tuberculosis reactivation in a patient with erythrodermic psoriasis under treatment with ustekinumab and a low dose steroid, despite isoniazid chemoprophylaxis. Eur J Dermatol 24: 508–509. doi: 10.1684/ejd.2014.2386 25120233 10.1684/ejd.2014.2386

[25] Saraceno R, Specchio F, Chiricozzi A, Sarmati L, Amicosante M, Chimenti MS, et al (2014) Usefulness of QuantiFERON^®^-TB Gold test in psoriatic patients under treatment with tumour necrosis factor blockers. Expert Opin Biol Ther 14: 151–156. doi: 10.1517/14712598.2014.860441 24303977 10.1517/14712598.2014.860441

[26] Sauzullo I, Mengoni F, Marocco R, Potenza C, Skroza N, Tieghi T, et al (2013) Interferon-γ release assay for tuberculosis in patients with psoriasis treated with tumour necrosis factor antagonists: in vivo and in vitro analysis. Br J Dermatol 169: 1133–1140. doi: 10.1111/bjd.12544 23909256 10.1111/bjd.12544

[27] Reddy M, Torres G, McCormick T, Marano C, Cooper K, Yeilding N, et al (2010) Positive treatment effects of ustekinumab in psoriasis: analysis of lesional and systemic parameters. J Dermatol 37: 413–425. doi: 10.1111/j.1346-8138.2010.00802.x 20536646 10.1111/j.1346-8138.2010.00802.x

[28] Feng CG, Jankovic D, Kullberg M, Cheever A, Scanga CA, Hieny S, et al (2005) Maintenance of pulmonary Th1 effector function in chronic tuberculosis requires persistent IL-12 production. J Immunol 174: 4185–4192. 15778379 10.4049/jimmunol.174.7.4185

[29] Tsai TF, Chiu HY, Song M, Chan D. (2013) A case of latent tuberculosis reactivation in a patient treated with ustekinumab without concomitant isoniazid chemoprophylaxis in the PEARL trial. Br J Dermatol 168: 444–446. doi: 10.1111/j.1365-2133.2012.11162.x 22816505 10.1111/j.1365-2133.2012.11162.x

[30] Chen DY, Shen GH, Chen YM, Chen HH, Hsieh CW, Lan JL. (2012) Biphasic emergence of active tuberculosis in rheumatoid arthritis patients receiving TNFα inhibitors: the utility of IFNγ assay. Ann Rheum Dis 71: 231–237. doi: 10.1136/annrheumdis-2011-200489 22021896 10.1136/annrheumdis-2011-200489

[31] Scrivo R, Sauzullo I, Mengoni F, Priori R, Coppola M, Iaiani G, et al (2013) Mycobacterial interferon-γ release variations during longterm treatment with tumor necrosis factor blockers: lack of correlation with clinical outcome. J Rheumatol 40: 157–165. doi: 10.3899/jrheum.120688 23204217 10.3899/jrheum.120688

[32] Mancuso JD, Bernardo J, Mazurek GH. (2013) The elusive "gold" standard for detecting Mycobacterium tuberculosis infection. Am J Respir Crit Care Med 187: 122–124. doi: 10.1164/rccm.201211-2033ED 23322793 10.1164/rccm.201211-2033ED

[33] Powell RD 3rd, Whitworth WC, Bernardo J, Moonan PK, Mazurek GH. (2011) Unusual interferon gamma measurements with QuantiFERON-TB Gold and QuantiFERON-TB Gold In-Tube tests. PLoS One 6: e20061 doi: 10.1371/journal.pone.0020061 21687702 10.1371/journal.pone.0020061 PMC3110578

[34] Chen YJ, Wu CY, Shen JL, Chen TT, Chang YT. (2013) Association between traditional systemic antipsoriatic drugs and tuberculosis risk in patients with psoriasis with or without psoriatic arthritis: results of a nationwide cohort study from Taiwan. J Am Acad Dermatol 69: 25–33. doi: 10.1016/j.jaad.2012.12.966 23375515 10.1016/j.jaad.2012.12.966

